# Atomic Resolution Interfacial Structure of Lead-free Ferroelectric K_0.5_Na_0.5_NbO_3_ Thin films Deposited on SrTiO_3_

**DOI:** 10.1038/srep37788

**Published:** 2016-11-25

**Authors:** Chao Li, Lingyan Wang, Zhao Wang, Yaodong Yang, Wei Ren, Guang Yang

**Affiliations:** 1 Electronic Materials Research Laboratory, Key Laboratory of The Ministry of Education& International Center for Dielectric Research, Xi’an Jiaotong University, Xi’an, China; 2 Frontier Institute of Science and Technology, State Key Laboratory for Mechanical Behavior of Materials, Xi’an Jiaotong University, Xi’an, China

## Abstract

Oxide interface engineering has attracted considerable attention since the discovery of its exotic properties induced by lattice strain, dislocation and composition change at the interface. In this paper, the atomic resolution structure and composition of the interface between the lead-free piezoelectric (K_0.5_Na_0.5_)NbO_3_ (KNN) thin films and single-crystalline SrTiO_3_ substrate were investigated by means of scanning transmission electron microscopy (STEM) combining with electron energy loss spectroscopy (EELS). A sharp epitaxial interface was observed to be a monolayer composed of Nb and Ti cations with a ratio of 3/1. The First-Principles Calculations indicated the interface monolayer showed different electronic structure and played the vital role in the asymmetric charge distribution of KNN thin films near the interface. We also observed the gradual relaxation process for the relatively large lattice strains near the KNN/STO interface, which remarks a good structure modulation behavior of KNN thin films via strain engineering.

Potassium sodium niobate (K_0.5_Na_0.5_NbO_3_, KNN), as an environment-friendly lead-free piezoelectric material, has been extensively studied since the earlier pioneering works by Saito *et al*.^1^. Most efforts have been made to improve the electrical properties of the KNN ceramics and thin films by constructing morphotropic or polytropic phase boundaries^2^,^3^,^4^,^5^,^6^,^7^. However, the satisfactory electrical properties have not been obtained for the KNN thin films. The new methods to further optimize the electrical properties of KNN thin films are still demanded. It is well known that the interface can significantly affect the performance of thin films due to the strain relaxation, charge trapping, inter-diffusion, and/or defect clustering. Tailoring the interface structure therefore could be an effective method to improve the electrical properties of thin films. Previous works of Kim *et al*.^8^ showed that the interesting switching properties and fatigue endurance of BaTiO_3_ thin films can be obtained by optimizing the interface structures. It was also shown that ferroelectric properties and leakage current behavior of BiFeO_3_ thin films were improved by tailoring the residual stress originated from the interface structures^9^,^10^. Moreover, atomic images of interface structure and polarization properties of PZT thin films were investigated by many researchers^11^,^12^,^13^,^14^. Lu *et al*.^15^ also found that a suitable interface structure can enhance the polarization of ferroelectric thin films.

For KNN thin films, the crystalline phase and electrical properties of textured KNN films have been reported but there exist few reports on the interface structure and its effect on the electrical properties^16^. In this work, we try to image the atomic scale interface structure of KNN thin films aiming at effectively modulating the performances by tailoring the interface structure. Since the epitaxial or textured thin films are favorable for structural investigations using electron microscopy, the highly textured KNN thin films were prepared by spin-coating a KNN precursor solution on single crystal strontium titanate (SrTiO_3_, STO) substrate. The microstructure of KNN/STO interface was investigated by the atomic resolution scanning transmission electron microscopy (STEM). The chemical composition, electronic structure, as well as the strain at the interface were analyzed by electron energy loss spectroscopy (EELS), First-Principles Calculations and the geometric phase analysis (GPA), respectively.

Figure 1(a) shows the atomic resolution high angle annular dark field scanning transmission electron microscopy (HAADF-STEM) image of the epitaxial KNN/STO interface along the [010] zone axis of STO. Since the intensity of an atomic column in HAADF-STEM image is approximately proportional to Z^2^ (Z: atomic number)^17^,^18^, the heavier atoms would show brighter spots in the HAADF-STEM image. Therefore, on the upper half of the interface (marked by the solid yellow rectangle) the bright spots represent Nb(O) atomic columns while the dim ones represent K(Na) atoms. For the same reason, the brighter and darker spots in the image of STO substrate are the Sr and Ti (O) atomic columns, respectively. Oxygen atoms are not visualized due to its small scattering cross-section in the HAADF-STEM image. A sharp KNN/STO interface is clearly observed and marked in Fig. 1(a). It is noted that the atomic columns in a monolayer at the interface (marked by solid yellow rectangle) show different contrast in comparison with the adjacent ones. The integrated intensity profile of this monolayer in Fig. 1(a) along the out-of-plane direction is given in Fig. 1(b). It shows the intensity of the interfacial monolayer is weaker than that of Nb(O) atomic columns in the KNN thin films but stronger than those of Ti(O) and Sr atomic columns in STO substrates. The contrast/intensity difference of this monolayer may indicate a chemical composition difference or the strain and dechanneling effects at the interface.

To determine whether the change of atomic composition leads to the contrast difference of the interfacial atomic columns in HAADF images, the atomic resolution electron energy loss spectroscopy (EELS) spectra imaging was used. The detailed acquisition of EELS spectra can be found in the Supplementary Information (SI). The EELS spectra were performed in the green rectangle region in Fig. 1(a). Color-coded elemental maps of Nb (red) and Ti (green) in the identical region are presented in Fig. 1(c) and (d), respectively. The corresponding composite map of Nb and Ti shows the relative position of Nb and Ti signals [Fig. 1(e)] and hence the location of the Nb and Ti atoms. With the help of the element distribution maps, we can experimentally confirm the coexistence of Nb and Ti atoms, and their occupation of B-site in the perovskite structure for the KNN/STO interfacial monolayer. Moreover, Fig. 1(f) presents the corresponding line profiles of the Nb and Ti signal intensity in Fig. 1(c) and (d), respectively. It intuitively shows that the Nb and Ti signal intensities at the interfacial monolayer are apparently weaker than those in the KNN thin films and the STO substrate, respectively. More detailed EELS spectra image is shown in Figure S2 of SI, which further confirms that Sr signal is only localized at A-site in STO (ABO_3_ structure) and no Sr signal can be observed at the B-site interfacial monolayer.

To quantify the ratio of Nb and Ti at the interfacial monolayer, the HAADF-STEM image simulation (WinHREM^*TM*^) was performed. In order to estimate the thickness of the sample, which will be used in the image simulation, zero loss peak and low loss spectra in the corresponding EELS spectra were also recorded and processed^19^,^20^,^21^. Using the formula *t* = *λln*(*I*_t_/*I*_0_) (where *t* is the specimen thickness, *λ* is the total bulk inelastic mean free path, *I*_*t*_ is the total area under the spectrum, *I*_0_ is the area under the zero-loss peak^22^) and taking the surface plasmon losses into account^23^, the thickness of the specimen is estimated to be about 25 nm. Moreover, the intensity of the HAADF images was also normalized relative to the incident probe intensity^22^,^24^. All experimental parameters used for the image acquisition were recorded and used as inputs for the image simulations. The values of the Debye-Waller factors were taken from the refs 25 and 26.

Figure 2(a) presents another similar experimental HAADF image of the KNN/STO interface. The interfacial monolayer is highlighted by the yellow frame. The average intensity of each type of atomic columns was measured and represented by the black quadrate in Fig. 2(b). For image simulations, the ratio of the Nb and Ti atoms in the monolayer was systematically adjusted until the intensity of atoms in the simulations matches with that of the experimental image. The final obtained Nb/Ti ratio of about 3/1 suggests that the KNN/STO interfacial monolayer contains about 75 at% Nb and 25 at% Ti atoms. The error bars in Fig. 2(b) represent the standard deviations from the mean intensity values of corresponding type atom columns in one image. A final simulated image is shown in Fig. 2(c).

The interfacial structure is known to be important for the performance of ferroelectric thin films. The changes in microstructure and chemical composition of the interfacial monolayer could induce the variation in charge distribution, chemical bonds and/or cause the formation of novel electronic structures at the interface. For achieving details, First-Principles Calculations were used to investigate the electronic structure of the interfacial monolayer. Figure 3(a) shows the simulation supercell, obtained from the experimental HAADF-STEM images. A slight tilting of oxygen octahedra is seen in the monolayer. Figure 3(b) and (c) show the diagrams of the slightly tilting oxygen octahedra and the in-plane atomic structure in the monolayer, respectively. In addition, it is found that the further from the interface the stronger the oxygen octahedral tilting (see Supplementary Figure S3). The suppression of lattice tilting in the monolayer could be attributed to the substrate restriction and the mixture of Ti in the KNN perovskite structure, because the bonding of Ti-O is shorter than that of Nb-O.

What is more, the substitution of Ti for Nb at the interfacial monolayer would lead to the change of charge transfer and bonding properties. For stoichiometric bulk KNN, the Fermi energy is located at the top of the valence band, formed by Nb 4*d* states, and indicates a typical insulator characteristics (see Supplementary Figure S4). It is known that the (K_0.5_Na_0.5_)O and NbO_2_ layers in KNN are charged with −1 and +1, respectively. However, the [001] stacking consists of SrO and TiO_2_ layers in STO, which are charge neutral. When the Nb^5+^ ions in the NbO_2_ layer bond directly to the oxygens in the SrO layer on the subsrate surface, the excessive Nb *4d*-state valence electrons will stay in anti-bonding orbitals and act as interfacial carriers. This would lead to a polar discontinuity at the KNN/STO interface and a higher leakage current density when an external electric field is applied. However, the polar discontinuity and leakge current enhancement are expected to be weakened for the interface with the mix of neutral TiO_2_ layer. The electronic structure evolution of the Nb/Ti mixed monolayer was characterized and shown in Fig. 3(d) with the partial density of states (PDOS) for atoms labelled in Fig. 3(c). It can be seen that the top of valence band of the monolayer is mainly formed by O 2*p* states, while the bottom of conduction band is composed of the partial Ti 3*d* states together with Nb 4*d*-state electrons. The Fermi energy at the KNN/STO interface is pushed into the conduction band, which suggests partial Nb 4*d* states and Ti 3*d* states electrons stay in antibonding states to act as the carriers at interface. Figure 3(d) shows there are multiple overlaps for the peaks of Nb 4*d* states and O 2*p* states, highlighted by the dashed lines. It indicates a strong and localized hybridization of Nb 4*d* states and their nearest neighbor O 2*p* states located at the low energy region of the valence band. Similarly, the hybridization between Ti 3*d* and O *2p* states in the full range of valence band is also seen in Fig. 3(d). The absence of Ti 4*s* and Nb 5*s* states at the valence band suggests that most Ti 4*s* and Nb 5*s* state electrons have been transferred to the O 2*p* states and formed ionic bonds.

Aiming to intuitively illustrate the variation of charge density, the charge density distribution at the interface is plotted in Fig. 3(e). It can be seen that the charge density around Nb and Ti atoms is connected with humps directed toward their neighboring O atoms. Moreover, the out-of-plane charge density between Ti and O atoms (marked by the white arrow) is lower than that between Nb and O (marked by the dark arrow) and this asymmetric distribution of out-of-plane charge density in the monolayer can be extended to a subsequent Nb-O bonds. This suggests the asymmetric charge distribution induced by Ti insertion at the interface can be maintained in the subsequent KNN thin films growth. To further verify the induced polar behavior with Ti substitutions, we present contour plots of the out-of-plane charge density difference along (001) plane in Fig. 3(f) that directly shows the charge transfer process during the bond formation. We can see that the outer-shell *d* and *s* states electrons of Nb and Ti atoms move to the 2*p* states of nearest neighbor O atoms, suggesting the formation of partial ionic bonding. Meanwhile, there is enhancement of the electronic density [the red region marked by the black arrow in Fig. 3(f)] in the region between Nb atoms and their nearest neighbor O atoms. The charge density accumulation is closer to the O atoms due to the larger electronegativity of oxygen, which implies the existence of polar covalent bonds. Compared to the notable charge accumulation between Nb and the nearest neighbor O atoms, there is no obvious charge accumulation between Ti and O atoms in the out-of-plane direction. It indicates a relatively stronger *p*-*d* coupling effect of the Nb-O bonds than Ti-O bonds. The asymmetric *p-d* coupling of Ti-O-Nb bonds influences the subsequent *p-d* coupling Nb-O-Nb bonds in the same direction, as shown in Fig. 3(f). The asymmetric electron distribution around Nb when bonding with the nearest O atoms in the out-of-plane direction will lead to a movement of Nb atoms in the oxygen octahedral, accompanied with the changed polarization. Different from the out-of-plane direction, the in-plane *p-d* coupling effect of Ti-O-Nb bonds shows relatively weaker asymmetry.

Furthermore, the interface monolayer with changed composition could also change the lattice parameter of the KNN thin films. As a result, an interficial strain field arises from the lattice misfit. Recent progress in digital image processing has enabled us to extract and quantify the lattice parameters with an high spatial resolution. The geometrical phase analysis (GPA) technique is considered as one of the most effective methods. It relies on the assumption that the image intensity peaks directly correspond to the positions of atomic columns in a given area^27^. Zhu *et al*.^28^ has reported that the HAADF-STEM images are more suitable for GPA technique than the phase-contrast-dominant high-resolution transmission electron microscopy (HRTEM).

Figure 4 shows the HAADF-STEM images of the cross-sectional KNN thin films with and without dislocations, as well as the corresponding GPA strain maps of *E*_*xx*_ (in-plane strain), *E*_*yy*_ (out-of-plane strain) and *E*_*xy*_ (shear strain). The scan distortions introduced during image acquisition have been corrected and the detailed procedure is given in SI. The *E*_*xx*_, *E*_*yy*_ and *E*_*xy*_ are relative values and represent local lattice displacements from the reference lattice. In this analysis, the STO substrate is used as reference, such as *E*_*xx*_ = (*a*^local^-*a*^sub^)/*a*^sub^ (*a* is the in-plane lattice parameter). The positive or negative value of *E* indicates the measured local lattice parameters being larger or smaller than the reference one, respectively. Figure 4(a)–(d) show the HAADF-STEM image and strain maps of an interface without dislocation. Using a profile across the interface with a width of 300 pixels (~14 nm), as shown in a rectangle, we show the relative lattice strains with the corresponding strain maps. The relative *a*-lattice strain does not show a dramatic overshoot near the interface, reflecting the actual interface perfection and no inter-diffusion. Across the interface, the relative *a*-lattice strain shows a gradual ascent in the KNN thin films till it reaches over 8 nm. Thus a lattice region over the 8 nm in Fig. 4(b) should be chosen for assessing the *a*-lattice spacing of the KNN thin films. The relative *a*-lattice strain is about 2.4% and the *a*-lattice of the KNN thin films is calculated to be about 0.400 nm. For the relative *c*-lattice strain *E*_*yy*_, it should be dramatically increased across the interface, accompanied by an obvious relaxation of *c*-lattice strain, due to the larger *c*-lattice parameter of KNN than that of STO. However, the relative *c*-lattice strain abnormally oscillates around zero, as shown in Fig. 4(c). One of the possible reasons is that the *c*-lattice strain of KNN thin films is small and just approaches to the inherent strain error in GPA digital processing^28^. Further studies on this point need to be conducted in the future. The oscillation around zero of *E*_*xy*_ near the interface in Fig. 4(d) indicates that there is little out-of-plane rotation in both the film and substrate, similar to the results of MgO/STO and STO/LAO interface^28^.

In our KNN thin films, the classic edge dislocations were also randomly observed at the interface. Figure 4(e), S5 and S6 in SI show the HAADF-STEM images of KNN/STO interface with the edge dislocations in different areas. The edge dislocation in the Fig. 4(e) is marked with the red rectangle. The scan distortion corrected GPA strain maps for the HAADF image in Fig. 4(e) are given in Fig. 4(f)–(h). It is clear that both relative *a*- and *c*-lattice strains instantaneously increase across the misfit dislocations, as shown in Fig. 4(f) and (g). The misfit strain at the dislocation can be up to 5.0%. The strong effect of dislocation on the shear strain is also observed, as shown in Fig. 4(h). In addition, the strain maps of more extensive area are given in Figure S7 and S8 in SI, showing the whole strain relaxation process near the interface.

In summary, a sharp epitaxial interface was observed between the KNN thin films and STO substrate. STEM-EELS analysis and HAADF image simulation indicated there is a B-site Nb and Ti cations mixture at the interfacial monolayer with a ratio of 3/1. The covalent and ionic mixture bonding nature was evaluated with First-Principles Calculations. The variation of the charge transfer and the enhanced polarization were also predicted as results of the asymmetric charge distribution around Nb atoms with the insertion of Ti into KNN perovskite lattice. We also investigated the interfacial lattice strains using GPA analysis and it was found that the interfacial strains can relax gradually at dislocation-free regions or abruptly via the dislocations.

## Methods

### Synthesis and Characterization

KNN thin films were prepared by a chemical solution deposition method and the single crystal SrTiO_3_ was used as the substrate. The details are shown in the Supplementary Information. The cross-sectional specimens were prepared using the tripod polishing method. The final thinning of the specimen was performed on a Gatan PIPS with liquid N_2_ cooling stage. The microstructure was characterized by scanning transmission electron microscopy (STEM) with Gatan Enfina electron energy loss spectroscopy (EELS) using JEOL ARM 200F electron microscope with probe spherical aberration corrector. In STEM mode a probe size of 0.1 nm, semi-convergence angle of *α* = 32 mrad and the collection angle of 80–170 *mrad* were used for HAADF imaging. The atomic resolution high-angle annular dark field (HAADF) image simulations were carried using the software package of WinHREM^*TM*^. The interface of KNN/STO geometry obtained from the HAADF-STEM image was used in First-Principles calculations, which were carried out using CASTEP code^29^. The electron-electron exchange and correlation effects were described by Perdew-Burke-Ernzerhof for solids (PBEsol) in generalized gradient approximation (GGA)^30^. Ultra-soft pseudo-potentials were utilized for the electron-ion interactions. In our calculations, cut-off energy of 370 eV and a 5 × 5 × 1 k-point Monkhorst Pack mesh in the Brillouin zone were used for the geometry optimization and the electronic structure calculation.

## Additional Information

**How to cite this article**: Li, C. *et al*. Atomic Resolution Interfacial Structure of Lead-free Ferroelectric K_0.5_Na_0.5_NbO_3_ Thin Films Deposited on SrTiO_3_. *Sci. Rep*. **6**, 37788; doi: 10.1038/srep37788 (2016).

## Supplementary Material

Supplementary Information

## Figures and Tables

**Figure 1:**
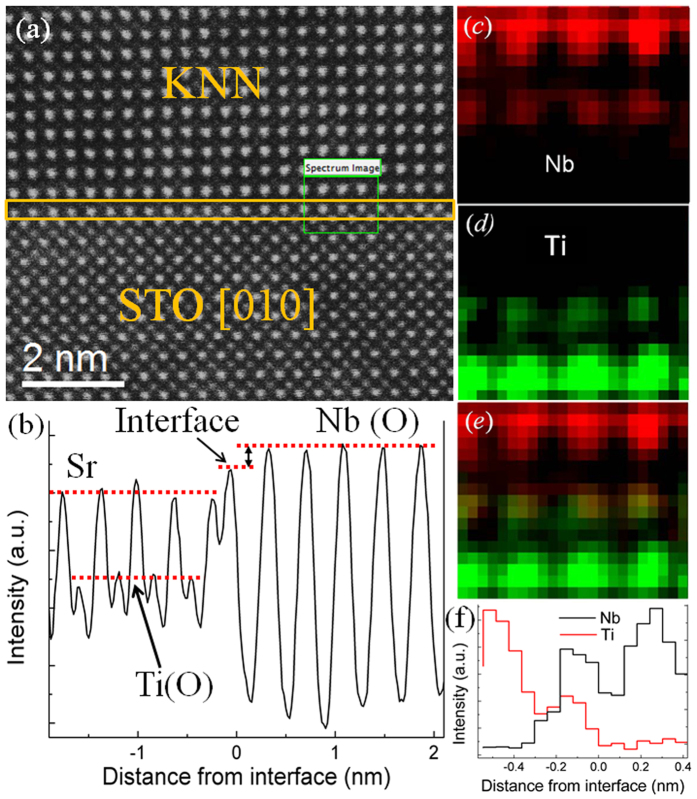
EELS spectra imaging of the KNN/STO interface (filtered). (**a**) HAADF-STEM image of KNN/STO interface with the area from which EELS mapping was carried out (highlighted with a green line rectangle); (**b**) integrated line profiles of atomic columns across the interface along the out-of-plane direction; (**c**) and (**d**) color-coded Nb and Ti elemental maps, respectively; (**e**) composite elemental map with Nb in red, Ti in green; (**f**) integrated line profiles of Nb and Ti signals in (**c**) and (**d**) across the interface, respectively.

**Figure 2:**
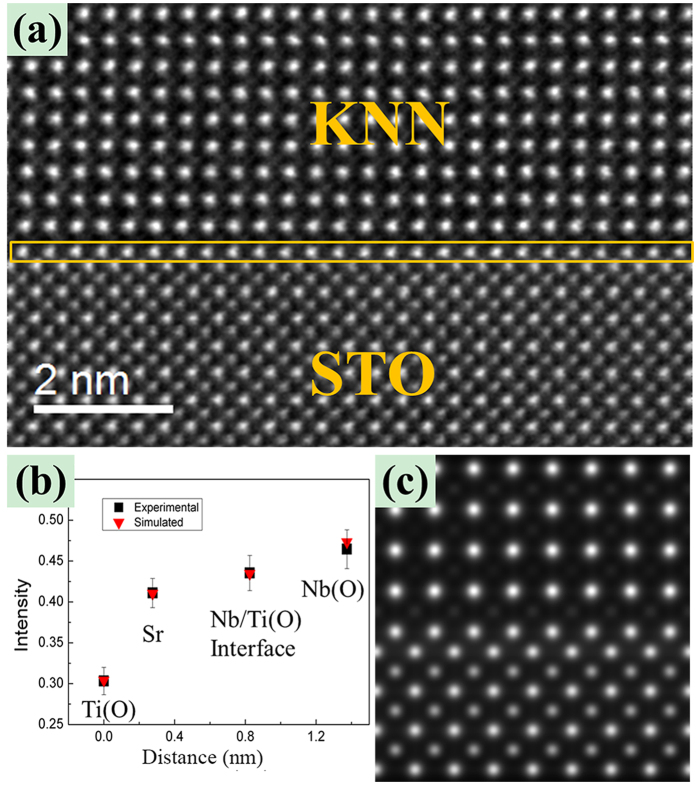
(**a**) HAADF image of KNN/STO interface, the interface is highlighted with the yellow rectangular, (**b**) the average intensity of each type atomic columns measured from the experimental image as represented by the black quadrate and the simulated corresponding intensities are represented by the red triangle (the atom columns at the interface are set to contain the mixture of 75% Nb and 25% Ti atoms), (**c**) simulated image of the interface.

**Figure 3:**
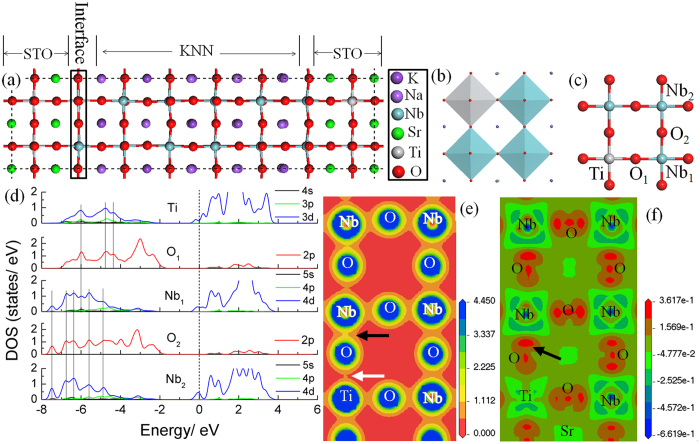
(**a**) Model of the KNN/STO interface, (**b**) diagram of oxygen octahedral, (**c**) the in-plane atomic structure of the KNN/STO interface, (**d**) the density of states of the interfacial atoms, (**e**) the contour plots of the out-of-plane charge density at the KNN/STO interface and (**f**) the charge density difference along a slice of the (001) plane for the interface layer. Positive and negative values indicate the accumulation and depletion of electronic charge, respectively.

**Figure 4:**
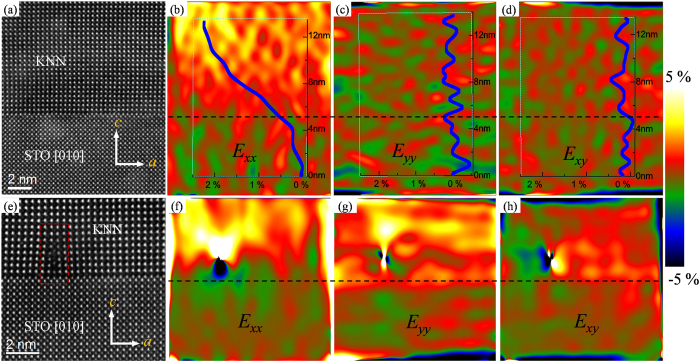
Strain mapping of the KNN/STO interface. (**a**) The atomic-resolution HAADF image of KNN/STO interface without dislocations; (**b–d**) the GPA *E*_*xx*_ (in-plane strain), *E*_*yy*_ (out-of-plane strain) and *E*_*xy*_ (shear strain) maps of (**a**), respectively; (**e**) the atomic-resolution HAADF image of KNN/STO interface with a dislocation; (**f–h**) the GPA relative *a*, *c*-lattice strain and shear strain maps of (**e**), respectively. The positive and negative values of *E* indicate the measured local lattice is larger and smaller than the reference lattice, respectively.
