# Supplementary material for: Atomic Resolution Interfacial Structure of Lead-free Ferroelectric K0.5Na0.5NbO3 Thin films Deposited on SrTiO3
Source: Sci Rep. 2016 Nov 25;6:37788. doi: 10.1038/srep37788 (PMC5122904; doi:10.1038/srep37788)
Supplement: Supplementary Information [file srep37788-s1.pdf]

Atomic Resolution Interfacial Structure of Lead-free Ferroelectric $K_{0.5}Na_{0.5}NbO_3$ Thin films Deposited on $SrTiO_3$

Chao Li[1], Lingyan Wang[1*], Zhao Wang[2], Yaodong Yang[2], Wei Ren[1]and Guang Yang[1]*

[1] Electronic Materials Research Laboratory, Key Laboratory of The Ministry of Education& International Center for Dielectric Research, Xi'an Jiaotong University, Xi'an, China

[2] Frontier Institute of Science and Technology, State Key Laboratory for Mechanical Behavior of Materials, Xi'an Jiaotong University, Xi'an, China

* l.y.wang@mail.xjtu.edu.cn, g.yang@mail.xjtu.edu.cn

Materials preparation

We fabricated KNN-based thin films by sol-gel processing. For the preparation of the KNN precursor solution, potassium acetate and sodium acetate were first dissolved in 2-methoxyethanol, and then the niobium ethoxide was introduced. 5.0 mol% manganese acetate and small amount of polyvinylpyrrolidone were subsequently added to the solution in order to obtain the KNN thin films with improved electrical properties according to the previous study. The stable precursor solution was deposited on [001]-cut STO substrates by spin-coating in ambient atmosphere. After deposition, the KNN gel film was dried at 200 degree Celsius for 2 min, pyrolyzing at 330 degree Celsius for 10 min, and annealing at 650 degree Celsius for 10 min in a

rapid thermal annealing furnace. The final KNN thin films with a thickness of about 200 nm were fabricated by repeating the coating-heat treatment process 4 times.

Image acquisition and simulation

In this work, HAADF-STEM was carried out using the aberration-corrected JEOL ARM 200F operated at 200 kV with a probe size of 0.1 nm, semi-convergence angle of α=32 mrad and a collection angle interval between 80-170 mrad. Before the atomic resolution HAADF images were recorded, two-fold astigmatism A_1, three-fold astigmatism A_2 and the axis coma B_2 were further adjusted by using the Ronchigram of anamorphous area close to the interesting area of the sample. HAADF image simulations were carried out in order to verify the contrast difference of the interface atomic columns. We used the (S)TEM simulation software WinHREM^{TM}. Thermal diffuse scattering was taken account in Weickenmeier-Kohl Scattering Factor. We inputted crystal structure and such real experimental parameters in microscopy as aberration coefficient in Cs-corrector, U_a=200kV, θ_{conv}=32mrad, θ_{HAADF}=[80-170] mrad. Δt_{slice} = 2Å, calculation step: 0.2Å. We just systematically varied the ratio of Ti and Nb atoms in crystal model of the interface.

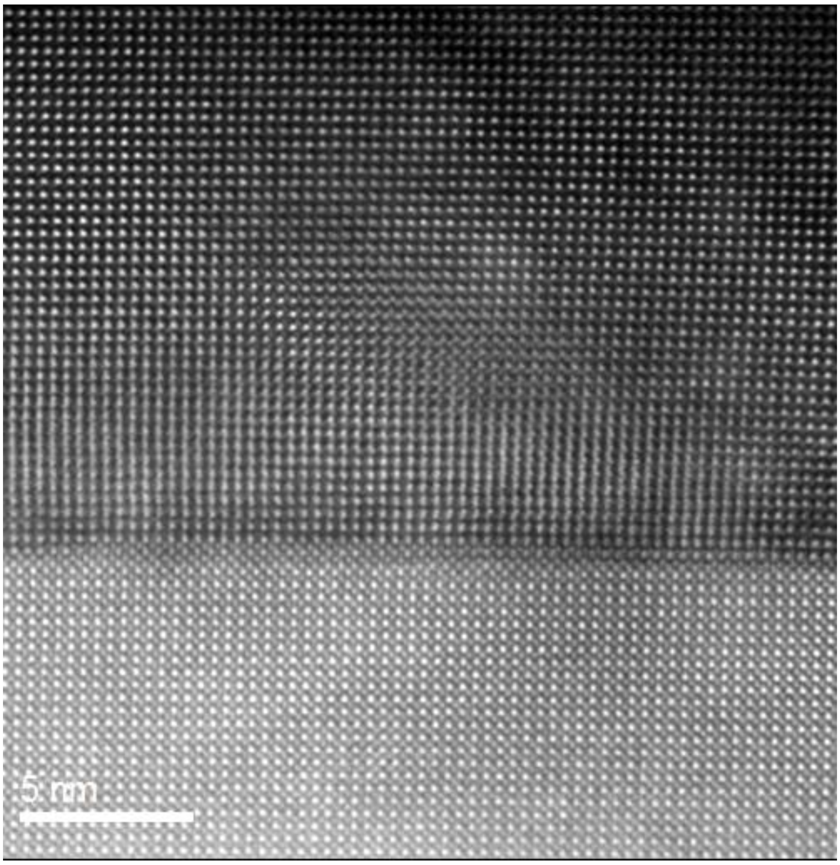

Figure S1. The atomic terrace at the interface.

EELS acquisition

For the EELS acquiring, 2cm camera length with 3mm EELS entrance aperture was used to maximize the intensity while achieve reasonable signal to noise ratio (SNR). The energy dispersion of 0.5 eV/ch was adopted to contain all the relevant edges, the scanning pixels step was 0.06 nm. After background subtraction of the signals, based on extrapolation of the background prior to the relevant edges, the intensity of the Nb $M_{2,3}$ (363 eV) edge and Ti $L_{2,3}$ edge (456 eV) signals were extracted. The corresponding color-coded Ti and Nb map showed the relative position of the Ti $L_{2,3}$ (green) and Nb $M_{2,3}$ (red) signals. In the EELS, the ratio of $\ln(I_t/I_0)$ is sensitive to thickness variation and proportional to t/λ, where I_t is the total area under the

spectrum, I_0 is the area under the zero-loss peak, λ is the total bulk inelastic mean free path and t is the specimen thickness. The plasmon scattering inelastic mean free path λ_B can be estimated to be 123.0 nm (for STO).

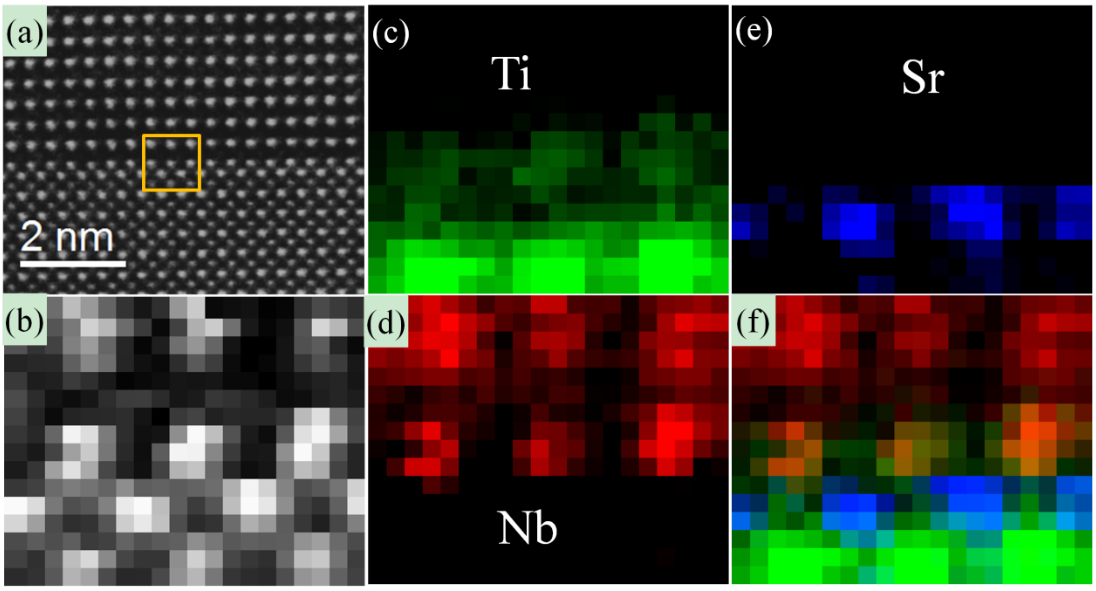

Figure S2. EELS spectra image of the KNN/STO interface. (a) HAADF-STEM image of KNN/STO interface with the area from which EELS image was carried out (highlighted with a solid line rectangle), (b) HAADF intensity recorded simultaneously while the EELS spectra in the region of interest [solid line rectangle in (a)] are acquired, (c), (d) and (e) false-colored Ti, Nb and Sr elemental maps, respectively, (f) combined elemental map with Ti in green, Nb in red and Sr in blue.

Density Functional Theory Modeling

The interface of KNN/STO geometry inferred from STEM was used in density functional theory (DFT) calculations, which were carried out using CASTEP code. The electron-electron exchange and correlation effects were described by

Perdew-Burke-Ernzerhof for solids (PBEsol) in generalized gradient approximation (GGA). Ultrasoft pseudo-potentials were utilized for the electron-ion interactions. In our calculation, cut-off energy of 370 eV and a $5\times5\times1$ k-point Monkhorst Pack mesh in the Brillouin zone were used for the geometry optimization and the electronic structure calculation. Electron correlation was taken into account with U_{eff} = 4.0 eV and 2.5 eV for Ti 3*d* and Nb 4*d* states, respectively. All atoms were allowed to relax until the force on each atom was below 0.01 eV/Å and the displacement of each atom was below 5.0×10^{-4}Å.

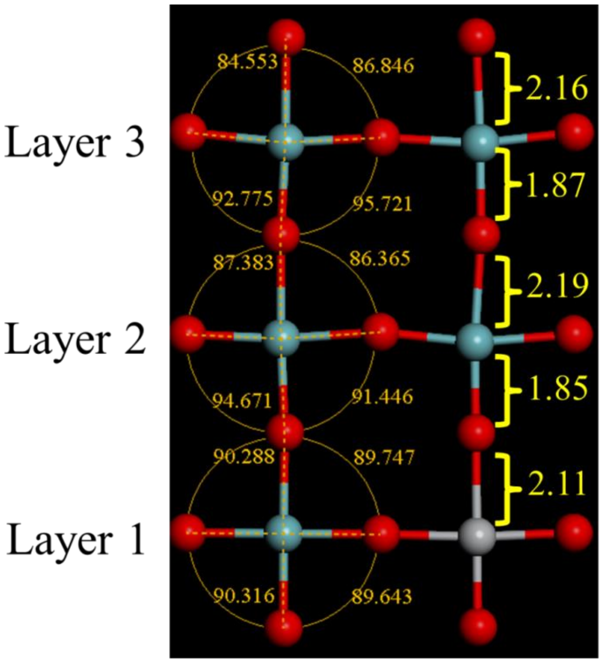

Figure S3. The simulated out-of-plane atom structure. The tilting of oxygen octahedra became stronger when far away from the intermixing monolayer (Layer 1).

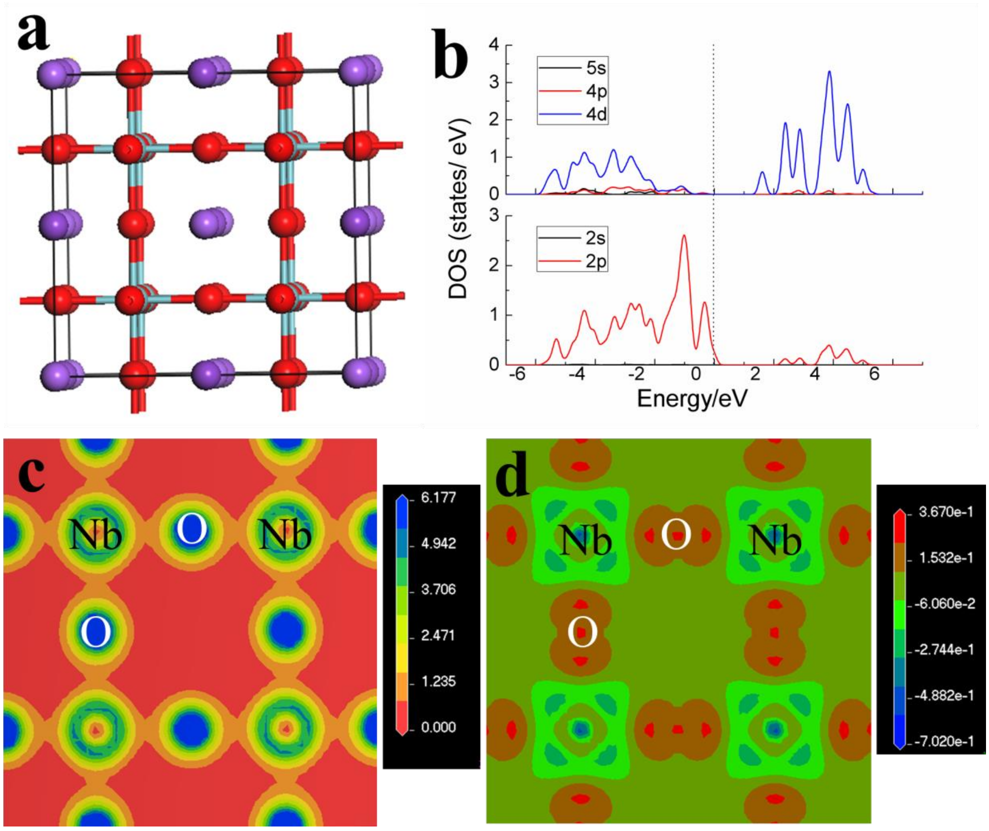

Figure S4. (a) The model of relaxed pure KNN; (b) the density of states of the interfacial atoms, where the Fermi energy is located at the top of valence band; (c) the contour plots of the charge density at the pure KNN and (f) the charge density difference at the pure KNN. Positive value indicates the accumulation of electronic charge; negative value indicates depletion of charge.

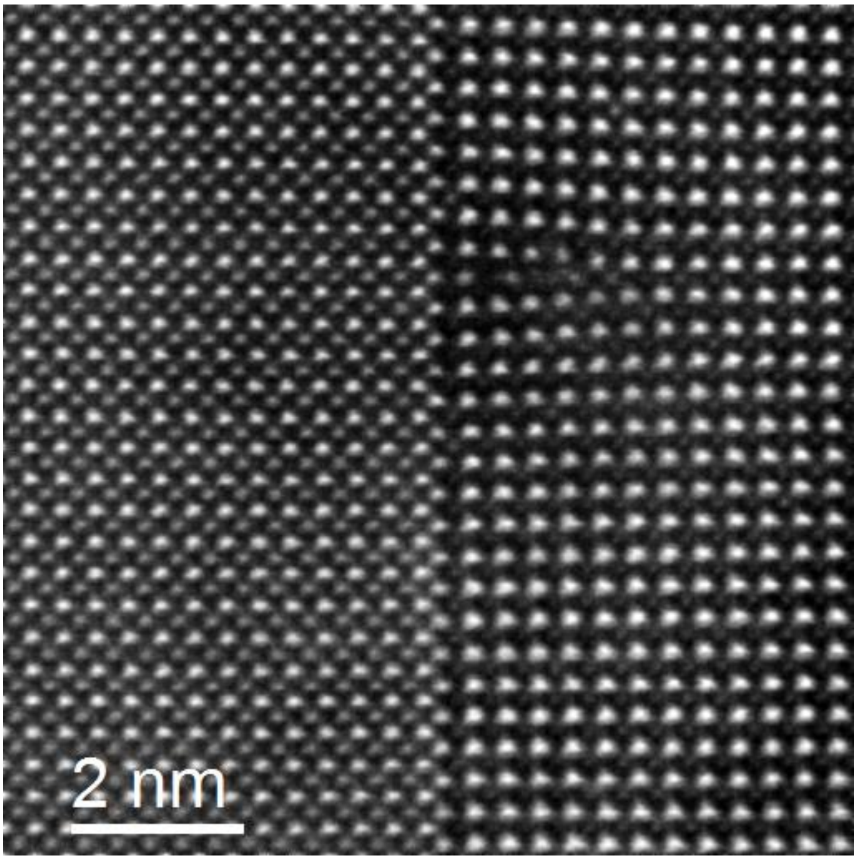

Figure S5. Atomic-resolution HAADF image of stress released KNN/STO interface with dislocation.

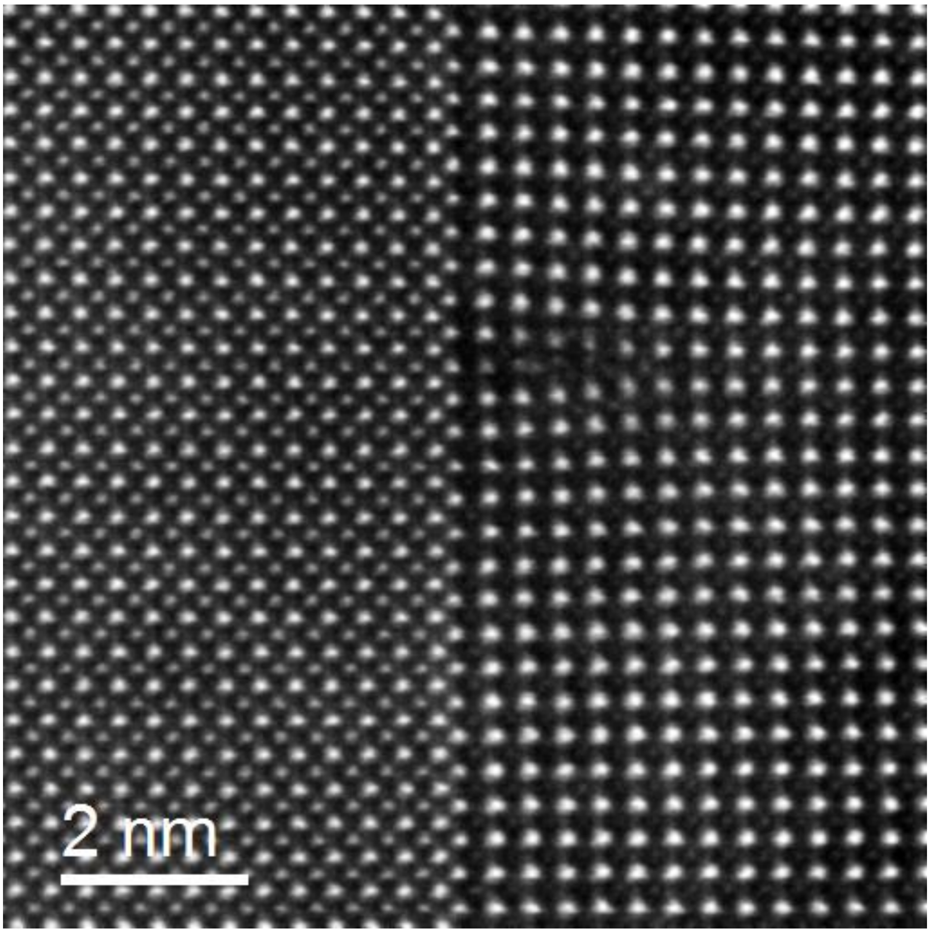

Figure S6. Atomic-resolution HAADF image of stress released KNN/STO interface with dislocation.

Scan distortion correction

The routine averages the phase horizontally (along the x-direction) within a ROI for the scan distortion correction. In detail, we used the ROI for the Define Reference for the scan distortion correction. The Extend ROI vertically option ensures that this is carried out from top to bottom of the whole image.

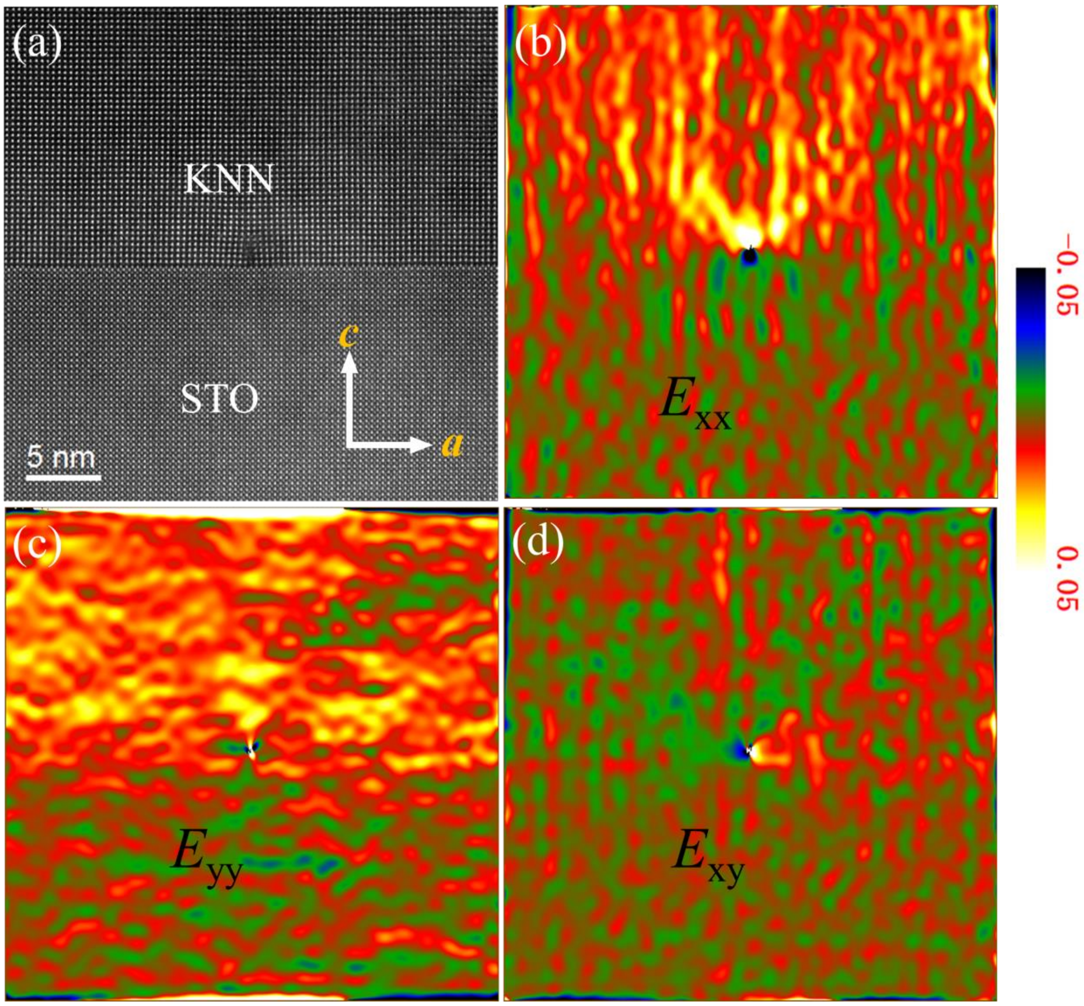

Figure S7. (a) An atomic-resolution HAADF image of KNN/STO interface, (b-d) the GPA E_{xx} (in-plane strain), E_{yy} (out-of-plane strain) and E_{xy} (shear strain) maps of (a), respectively. The positive and negative values of E indicate the measured local lattice is larger and smaller than the reference lattice of STO, respectively.

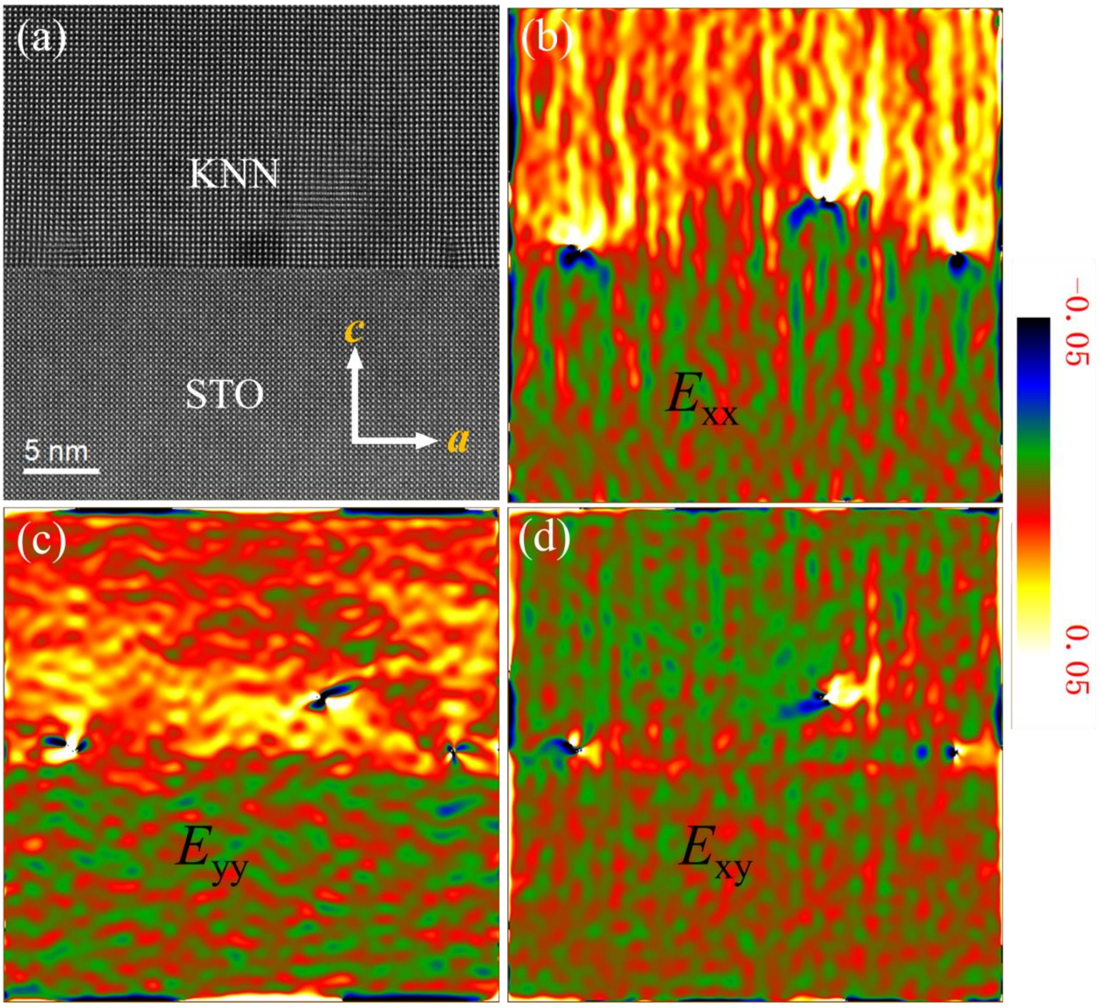

Figure S8. (a) An atomic-resolution HAADF image of KNN/STO interface, (b-d) the GPA E_{xx} (in-plane strain), E_{yy} (out-of-plane strain) and E_{xy} (shear strain) maps of (a), respectively. The positive and negative values of E indicate the measured local lattice is larger and smaller than the reference lattice of STO, respectively.
